# Respect and admiration

**Published:** 2018

**Authors:** Victor Lorin Purcarea

**Affiliations:** * “Carol Davila” University of Medicine and Pharmacy, Bucharest

**Fig. 1:**
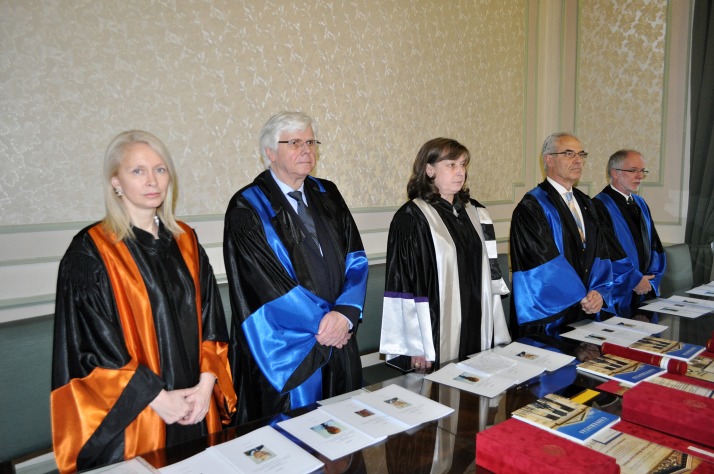
From left to right: Assoc. Prof. Paula Perlea, MD, PhD; Prof. Gottfried Schmalz, MD, PhD, med. dent.; Prof. Ecaterina Ionescu, MD, PhD; Prof. Ioannis Iatrou, MD, PhD; Prof. Aaron Palmon, MD, PhD, med. dent.

The imposing series of personalities from the whole world, who were awarded the “Doctor Honoris Causa” title by “Carol Davila” University of Medicine and Pharmacy in Bucharest, the biggest and oldest University of Medicine in Romania, opened in 1966 by Prof. A. Butendandt from Max Planck Institute in Germany and Laureate of Nobel Prize, continued with three great academic personalities in the field of Dentistry (**Fig. 1**).

**Fig. 2:**
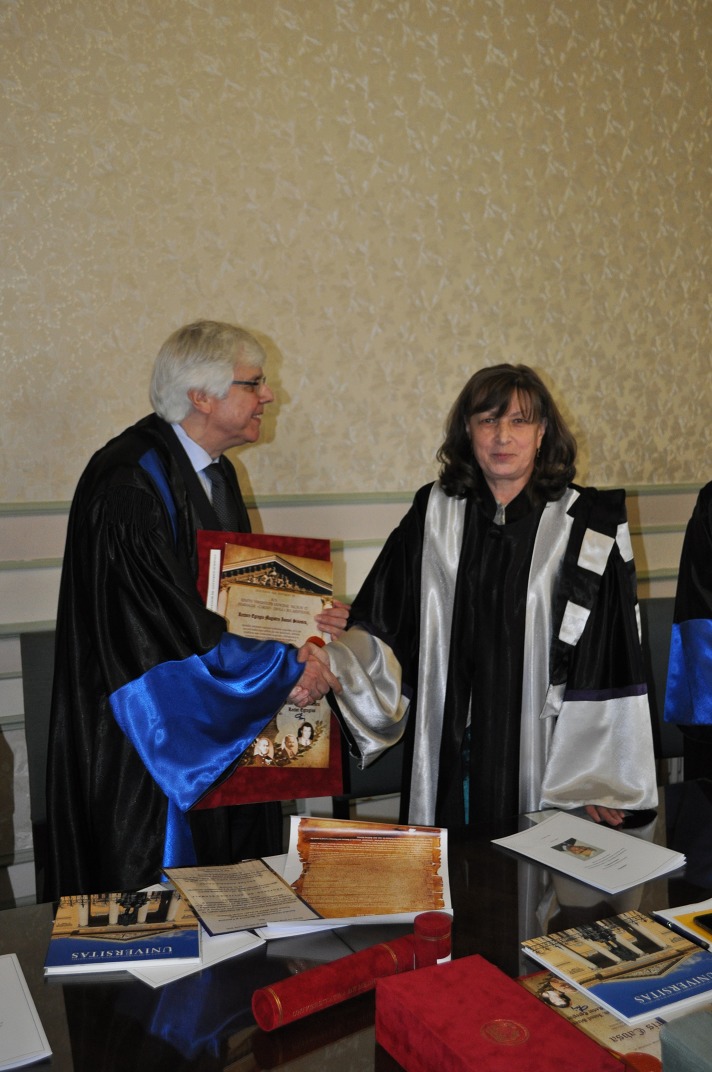
Prof. Gottfried Schmalz, MD, PhD, med. dent. and Prof. Ecaterina Ionescu, MD, PhD

*„Science is organized knowledge. Wisdom is organized life”. Kant’s quotes provide us an adequate context to talk about a savant who offers wisdom through science. Mentioning his merits today, in order to justify the reason for which we award Professor Schmalz the highest distinction from an academic institution, is a delicate endeavour, taking into account the difficulty to make a choice among the multitude of achievements, research fields and recognitions* – were the starting words of the inspired “Laudatio” in the honor of distinguished Professor Gottfried SCHMALZ, MD, PhD, Professor in the Department of Operative Dentistry and Periodontology at the University of Regensburg, Germany and guest Professor at the Dental School of the University of Bern, Switzerland. Prof. Schmalz, MD, PhD obtained his D.D.S degree (“Doctor of Dental Surgery”) from the University of Bonn, Germany, in 1971, and in 1972 his DMD (“Doctor of Dental Medicine”) also in Bonn. He has been Chairman of the Department of Operative Dentistry and Periodontology at the University of Regensburg, Dean of the Faculty of Medicine of the University in Regensburg, and, for more than 20 years, Dean of the Faculty of Dental Medicine of the same university. Moreover, he has been member and President of the Senate of the University in Regensburg (**Fig. 2**).

He has been President of several national dental associations such as German Association for Operative Dentistry (DGZ), German Scientific Society for Dentistry (DGZMK) and German Association for Professors in Dentistry (VHZM). He has later been elected Honorary Member of these associations.

**Fig. 3:**
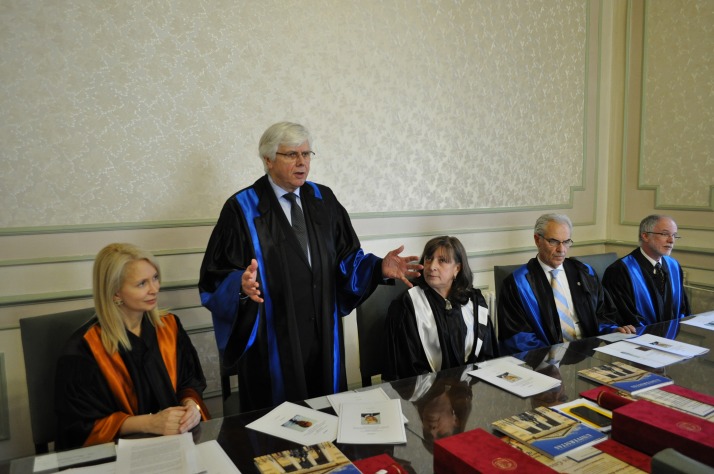
Prof. Gottfried Schmalz, MD, PhD, med. dent., University of Regensburg, Germany

At the international level, Prof. Schmalz, MD, PhD has been elected President of the Continental European Division of the International Association for Dental Research (IADR) as well as for the Pan European Region (including Israel) of the same association. He was also President of the Pulp Biology and Regeneration group of IADR (**Fig. 3**).

**Fig. 4:**
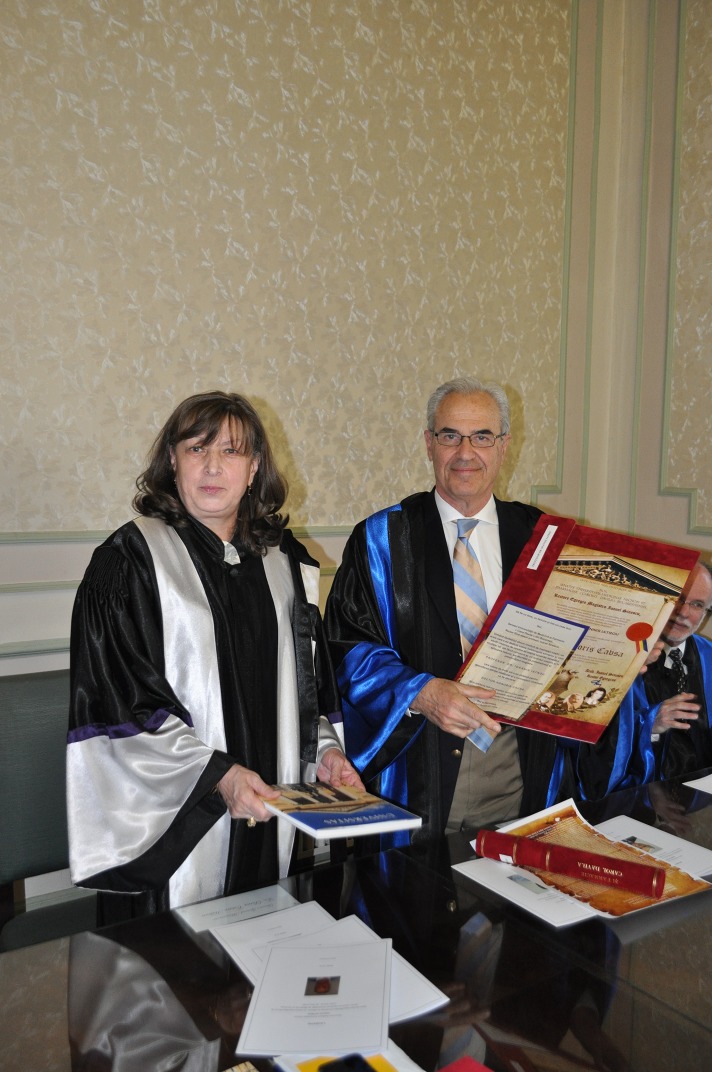
Prof. Ecaterina Ionescu, MD, PhD and Prof. Ioannis Iatrou, MD, PhD

Prof. Schmalz, MD, PhD has published more than 250 articles in PubMed listed journals, with a Hirsch index of 24, and over 1800 citations. For his scientific work, he received more than 35 prices and awards, among which the “European Award for Substitution of Animal Experimentation”, Anny Eck-Hieff Prize for the “Development of an in-vitro pulp chamber system for toxicity testing of filling materials in dentistry”, “Distinguished Scientist Award” for his achievements in Pulp Biology Research, “Distinguished Service Award”, and “Award of Excellence” of the European Federation of Conservative Dentistry. He is Adjunct Senior Scientist at the Houston Center for Biomaterials and Biomimetics, University of Texas and Member and Senator of the German National Academy of Sciences. Since 2014, he has been guest Professor at the Dental School of the University of Bern, Switzerland. In 2015, he received the Doctor Honoris Causa (Dr. h.c.) distinction from “Iuliu Hatieganu” University in Cluj-Napoca, Romania (**Fig. 4**).

Professor Ioannis IATROU, MD, PhD was elected Professor of Oral and Maxillofacial Surgery (OMFS) at the Dental School of the National and Kapodistrian University of Athens, in 2012.

He was member of the Senate of the same University (for five periods) and became Head of the OMFS Department during 2014-2016. During 1999-2016, he was Director of the OMFS University Clinic at the "A. Kyriakou" Children's Hospital of Athens having performed more than 1500 major surgeries of the maxillofacial regions in Children and Adolescents mainly including fractures, benign and malignant odontogenic and non-odontogenic tumors, arteriovenous malformations, clefts and syndromes involving the maxillofacial regions. He was also acting as Director of the post-graduate program on ”Dentoalveolar Surgery”, Dental School, University of Athens (2012-2017).

**Fig. 5:**
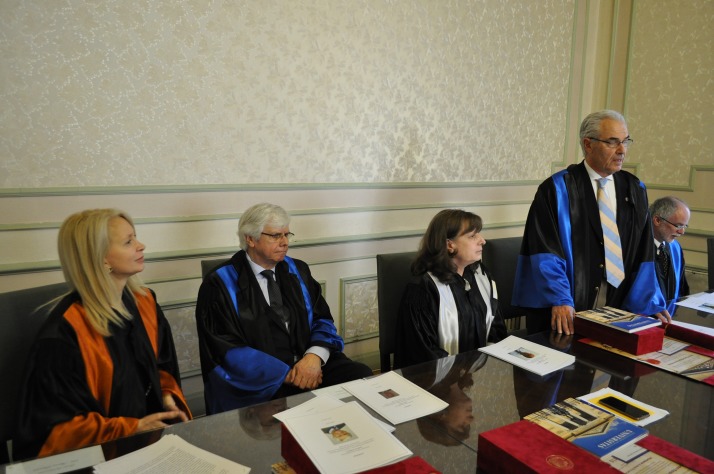
Prof. Ioannis Iatrou, MD, PhD, Dental School of the National and Kapodistrian University of Athens, Greece

He has also been President of the Hellenic Association of Oral and Maxillofacial Surgery (2006-2008 and 2014-2017) and Editor-in-Chief of its official Journal, "Archives of the Hellenic Association of Oral and Maxillofacial Surgery". He is member of the European Association for Craniomaxillofacial Surgery, Councilor for Greece (2010-2016), member of the Editorial Board and reviewer of its official Journal (**Fig. 5**).

**Fig. 6:**
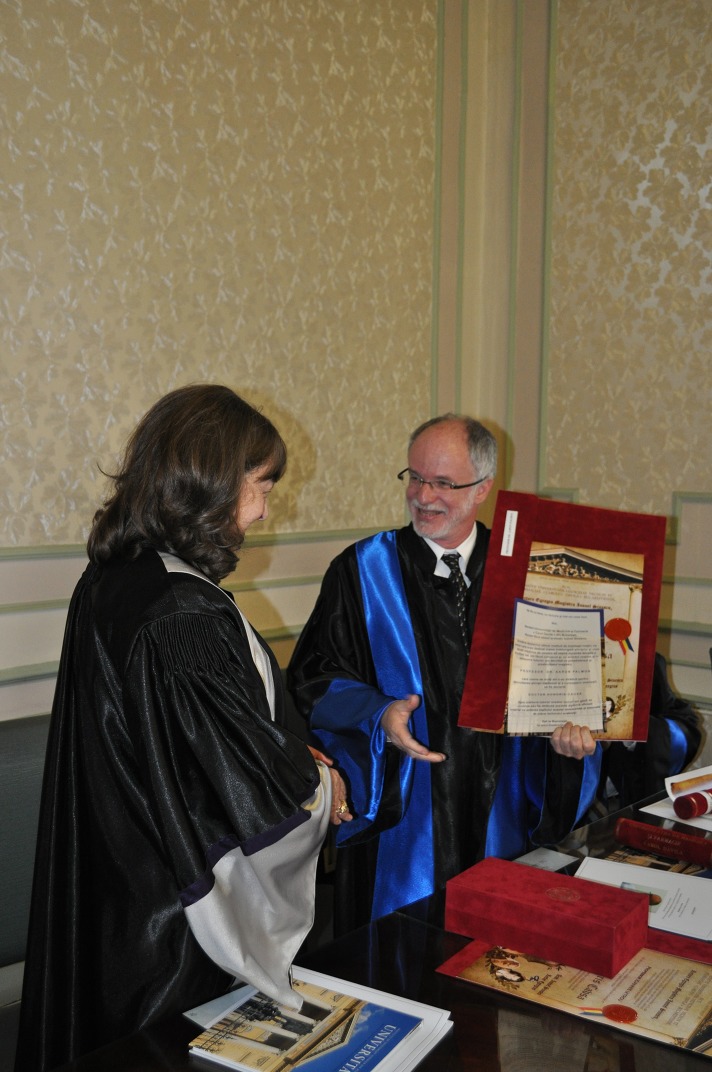
Prof. Ecaterina Ionescu, MD, PhD and Prof. Aaron Palmon, MD, PhD, med. dent.

He has also been honorary guest speaker in 14 European countries (Albania, Austria, Bosnia-Herzegovina, Belgium, Bulgaria, Denmark, Germany, France, Italy, Kosovo, Netherlands, Romania, Serbia, and Turkey). He is founding member of the Strasburg Osteosynthesis Research Group (SORG), a leading international scientific Organization with state of art statements concerning treatment of skeletal diseases of face and skull. Recently (2017), his team won the first prize of this group, concerning a research project dealing with syndromic and non-syndromic children treated with mandibular distraction osteogenesis. His main fields of interest are maxillofacial traumatology and oncology for children, orthognathic surgery, clefts, distraction osteogenesis, implantology, and oral surgery (**Fig. 6**).

Professor Aaron PALMON, MD, PhD, is a Professor in the Institute of Dental Sciences at the Hebrew University, Jerusalem, Israel and trained at the Hebrew University, where he received his "Doctor of Dental Medicine" diploma.

Prof. Palmon’s area of interest in research focused on molecular medicine of salivary glands. He developed technologies for gene transfer for salivary glands and, in recent years, concentrated on culturing salivary gland cells including salivary gland stem cells. Each year, 500,000 new cases of head and neck cancer occur worldwide. Most of these patients lose their salivary gland function following irradiation therapy. They suffer significant morbidities and their quality of life is greatly compromised. Prof. Palmon, MD, PhD isolated salivary stem cells that were able to restore saliva production in head and neck irradiated animal models, for future therapy of head and neck irradiated cancer patients. Prof. Palmon also developed technologies promoting the diagnostic value of Oral Fluids (OF) for both local and systemic diseases. Specifically, he developed a new toolbox to unmask "hidden" proteins in saliva, to concentrate and increase signal intensity of protein biomarkers using traditional lateral flow immunoassay tests as a platform for OF rapid diagnostic tests.

**Fig. 7:**
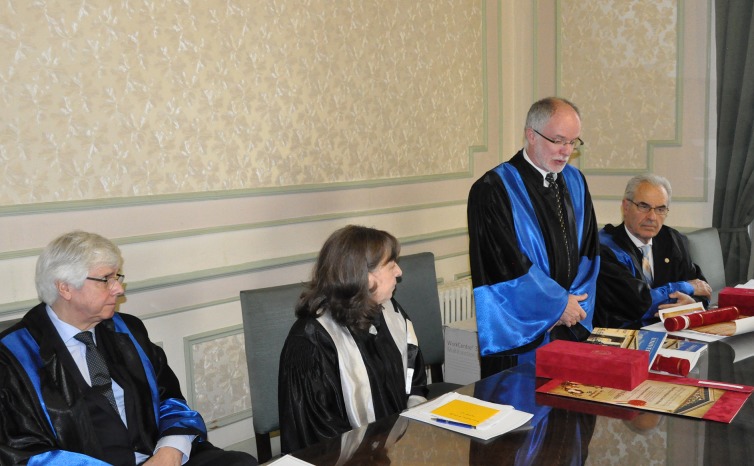
Prof. Aaron Palmon, MD, PhD, med. dent., Institute of Dental Sciences at the Hebrew University, Jerusalem, Israel

His work was supported by many research agencies (more than 30) including the Israeli Science Foundation, United States-Israel Binational Science Foundation, German-Israel Science Foundation, Ministry of Sciences, and Ministry of Health. His work was published in many international leading journals and awarded by many prizes. Prof. Palmon, MD, PhD has served as the president of the Israeli Division of the IADR, Director of the Graduate Program in Bio-Medical Dental Sciences and the Chairman of the Institute of Dental Sciences at The Hebrew University (**Fig. 7**).

**Fig. 8:**
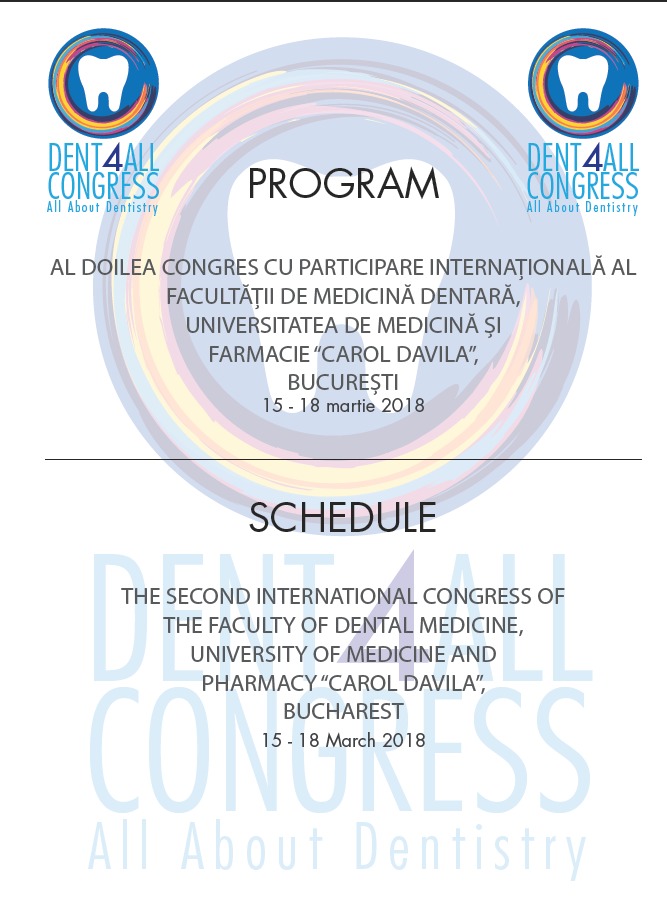
Cover of the Second International Congress of the Faculty of Dental Medicine, "Carol Davila" University of Medicine and Pharmacy, Bucharest

Within the university system, Prof. Palmon engaged in the administrative leading. Prof. Palmon, is currently serving as the Dean of the Hebrew University-Hadassah, Faculty of Dental Medicine, for a second term, and is the founder and director of the Hebrew University Teaching and Learning Center, the director of MEITAL, which is the Israeli Inter-University Center for e-Learning and management member of the Israeli university forum of teaching and learning centers (**Fig. 8**).

The event took place on 15-18 March 2018 in the imposing Boardroom of the Rectorate of "Carol Davila" University of Medicine and Pharmacy in Bucharest, during the second edition of the International Congress of Dental Medicine, dedicated to the 70 years jubilee from the founding of the Dentistry Faculty.

**Executive Editor Professor Eng. Victor Lorin Purcarea, PhD**

